# Evaluation of the risk of lung cancer associated with NAD(P)H: quinone oxidoreductase 1 (*NQO1*) C609T polymorphism in male current cigarette smokers from the Eastern India

**DOI:** 10.22099/mbrc.2020.36467.1481

**Published:** 2020-09

**Authors:** Santanu Banerjee

**Affiliations:** Department of Biotechnology and Dr B C Guha Centre for Genetic Engineering and Biotechnology,University College of Science and Technology, University of Calcutta, Kolkata -700019, India

**Keywords:** NQO1, NSCLC, SNP, Cigarette smoke, Quinone

## Abstract

NAD(P)H: quinone oxidoreductase 1 (NQO1) is an endogenous cellular defence mechanism against several carcinogenic quinones derived from cigarette smoke. *NQO1* C609T polymorphism is a strong determinant of NQO1 structure and function. The people with mutant allele for this polymorphism has significantly reduced NQO1 activity. In this study, we tried to evaluate the risk of lung cancer associated with this polymorphism in male current smokers of the Eastern India. Using PCR-RFLP method, we compared the *NQO1* C609T genotype distribution in male current smokers with (n=150) and without (n=200) lung cancer. We observed significant variation of genotypic distribution between these two groups. The allele frequency of the variant C609T allele were 40.3% and 32.7% in smokers with and without lung cancer, respectively. From the genotypic comparison between the two smoker groups, it was found that a higher risk (OR=1.64, 95% CI: 1.05-2.55, P<0.05) of lung cancer was associated with *NQO1* C609T polymorphism.

## INTRODUCTION

NAD(P)H: quinone oxidoreductase 1 (NQO1) can detoxify benzoquinones by reducing it to usually lesser toxic hydroquinones in a single two-electron step [1]. It is effective against several cigarette smoke-derived quinone components such as *para*-benzoquinone [2] and benzo (a)pyrene 3,6-quinone [3]. Although a number of polymorphisms have been found for the NQO1 gene, a particular single nucleotide polymorphism (SNP) found at the 609 position in *NQO1* cDNA has profound phenotypic consequences [4] on its stability and function. This polymorphism is a C to T change at position 609 of the cDNA which codes for a proline to serine change in the sequence of the human protein. The altered protein from NQO1*2 allele is observed to be less stable and undergoes rapid ubiquitination and degradation by proteasomal pathway [5]. Individuals with NQO1*2 homozygous genotype have virtually undetectable NQO1 protein [1]. 

In 2015, almost 1.7 million cancer-related deaths were attributed to lung cancer [6]. Cigarette smoking has been established as the single major cause of lung cancer by extensive epidemiological studies [7]. Cigarette smokers show variable susceptibility to lung cancer. This is mostly due to smoker’s variable ability to metabolize carcinogenic compounds derived from cigarette smoke [8, 9]. Several previous studies strongly indicated the association of the variant *NQO1 *C609T genotypes with lung cancer [10, 11]. In a case-control study, it has been indicated that the presence of NQO1*2 allele may be associated with the risk of non-small cell lung carcinoma (the major type of lung cancer) in Indian population [12]. As the *NQO1* genotypes vary widely among different parts of our nation [12-14], it was worthy to check *NQO1* C609T status in smokers with or without lung cancer in the Eastern India.

## MATERIALS AND METHODS

**Study design: **The risk associated with *NQO1* C609T status was evaluated in the following two groups of male smokers: 200 smokers without lung cancer and 150 smokers with lung cancer (NSCLC). As the prevalence of cigarette smoking is much higher in men than in woman in India [15], we confined our study to male current smokers ranging in age from 45 to 75 years. In general, any person having uncontrolled diabetes, malnutrition, known HIV seropositive status, or taking immunosuppressive drugs or on systemic steroid therapy were excluded. 

For the smokers without lung cancer group, male smokers without history of any kind of cancer and other cigarette smoke (CS)- induced diseases were included in the study. Smokers without CS-induced diseases and smokers with lung cancer were recruited from the Department of Medical Oncology, Medical College, Kolkata. Total one hundred and fifty individuals were genotyped for *NQO1* C609T status in this group (smokers with lung cancer). A detailed questionnaire was completed for each individual to provide information on the age, mean of daily cigarette consumption, and the number of years of smoking. Samples were collected over four years (2012-2016). This study involving smokers with or without Lung cancer was approved by ethical committee of Medical College of Kolkata. It was also approved by Institutional Bioethics Committee for Human Research Studies, University of Calcutta. Informed consent was obtained from all participants.

Venous blood (3ml) was collected from all study group individuals after acquiring their informed consents. Whole blood and serum were transported in ice-cold condition to the Biotechnology laboratory of the University of Calcutta on the same day. 

**Genotyping: **Genomic DNA of human blood was isolated using QIAGEN’s Blood Mini DNA Isolation Kit according to the manufacturer’s protocol. Specific primers [16] were used to amplify *NQO1* gene from human blood by QIAGEN’s Fast Cycling PCR kit according to the manufacturer’s protocol. The PCR product was purified by QIAGEN PCR purification kit as per the manufacturer’s protocol. 

The purified PCR product was digested using Hinf1 enzyme (from Sib enzyme). The 230 bp long PCR product from a wild-type *NQO1* allele contains a single HinfI restriction site (5'-GANTC-3'). Upon restriction digestion (Hinf1), it yields two fragments with 195bp and 35bp in length. On the other hand, 230bp long PCR product from an *NQO1* allele containing the C609T SNP has two HinfI restriction site due to a C to T conversion. Upon digestion, it yields three fragments of 151bp, 44bp, and 35 bp. This characteristic feature of *NQO1* gene is used for genotyping. The 195 bp and 151 bp fragments are visible under ultraviolet light. 

**Statistical analysis: **A goodness-of-fit χ2 test was performed to evaluate whether the polymorphisms were in Hardy–Weinberg equilibrium for the two groups. Cochran Armitage (CA) Trend test was used to compare the genotype distribution between the two smoker groups. For the evaluation of disease-risk odds Ratio, relative risk and 95% confidence interval (CI) were measured. Cochran Armitage (CA) Trend test was performed for evaluation of linear trend in additive, dominant and recessive model. All statistical analysis was performed by MedCalc and XLSTAT software. Comparisons between the mean ranks of the general attributes of the two smoker groups were performed by Mann-Whitney U test in SPSS software. 

## RESULTS

The mean (± SD) ages of the two groups were 58.7±7.9 (smokers without NSCLC) and 60.3 ± 9.1 (smokers with NSCLC). The mean rank of age for smokers with lung cancer (275.54) was greater than that of the smokers without the disease (247.09). Mann Whitney U test revealed that there was no significant difference (P>0.05) of age between these two groups. The mean (± SD) pack-years of smoking of the two groups were 38.4 ± 17.7 (smokers without NSCLC) and 39.6 ± 11.4 (smokers with NSCLC). The mean rank of pack-years of smoking for smokers with lung cancer (306.42) was greater than that of the smokers without the disease (269.32). Mann Whitney U test revealed that there was significant difference (P<0.05) of pack-years of smoking between these two groups.

Genotypic distributions of the two groups were in Hardy Weinberg equilibrium (smokers without NSCLC: χ^2 ^=1.23, P>0.05; smokers with NSCLC: χ^2^ =3.36, P>0.05). Table 1 describes the actual number of individuals in the three different genotype groups (CC, CT and TT) and the allele frequencies (wild type & variant allele) of *NQO1* C609T from the two smoker groups. The allele frequency of the variant C609T allele were 40.3% and 32.7% in smokers with and without lung cancer, respectively. 

**Table 1:** *NQO1* C609T genotype distribution among smokers with or without lung cancer

**Type**	N		***Genotypes***		**Allele frequency**	
		CC	CT	TT	p	q
Smokers without lung cancer	200	87	95	18	67.3	32.7
Smokers with lung cancer	150	48	83	19	59.7	40.3

N, number of subjects in each group.

Cochran-Armitage-linear trend-test (CA trend test) were performed to evaluate the difference of genotypic distribution between the control group and the diseased group. The test revealed that the genotypic distribution of three genotypes of *NQO1* C609T (i.e., CC, CT and TT) has significant variation (CA test χ^2^= 4.77, P<0.05) between the two smoker groups. Cochran–Armitage trend test was also performed for analysis of genotype variation in additive, dominant [(TT+CT) vs CC] and recessive model [TT vs (CT+CC)]. For smokers with lung cancer, significant (P<0.05) variations were observed in both additive and dominant model. For the dominant genetic model, the odds ratio and relative risk were 1.64 (95% CI: 1.05-2.54, P<0.05) and 1.33 (95% CI: 1.02-1.74, P<0.05), respectively. Table 2 describes the odds ratio associated with heterozygous and homozygous variantsfor *NQO1* C609T.

**Table 2:** Risk of NSCLC associated with NQO1 C609T variant genotypes in male current smokers

**Statistical parameters**		**Variant genotypes for NQO1 C609T SNP**	
		Heterozygous (CT)	Homozygous variant (TT)
Odds Ratio	OR	1.58	1.91
	95% CI	1.00-2.50	0.92-3.98

## DISCUSSION

The protective role of NQO1 against several CS-derived carcinogenic quinones is well established. This explains the higher risk of developing lung cancer in smokers in the presence of *NQO1* C609T polymorphism [11, 12, 17]. An alternative point of view regarding the role of NQO1 is also considered in scientific studies. Some researchers have found that NQO1 can activate some environmental or CS- derived procarcinogens [18-20]. Thus, some believe that fully active NQO1 can increase the risk of cancer. Hamajima et al, suggested that the two variant genotypes (CC & TT) of this particular SNP might be associated with the variable risk of developing lung cancer [10].

The genotypic distribution of this *NQO1* C609T polymorphism varies widely across different ethnicity. The studies from India also revealed that the distribution of the genotypes is different in different parts of India [12-14]. All these facts and findings encouraged us to evaluate the role of *NQO1* C609T polymorphism in CS-related lung cancer (NSCLC) in the Eastern India.

In our study, we observed that the smokers with lung cancer (NSCLC) have a significantly higher pack-years of smoking compared to the smokers without the diseases. The genotypic distribution of *NQO1* C609T in the Eastern India is different from other regions of the country. Smokers with lung cancer vary significantly in genotypic distribution from the control group. The C609T allele frequency is significantly higher in the smokers with lung cancer in comparison to the smokers without the disease. Our present study on the male current smokers from the Eastern India indicated that the risks of developing lung cancer in association with *NQO1* C609T was significant and the odds ratio was greater than one. We recommend further validation of this finding using larger sample size from this particular region of the country by independent studies. 
